# “Stealth Scripts”: Ultrashort Pulse Laser Luminescent Microscale Encoding of Bulk Diamonds via Ultrafast Multi-Scale Atomistic Structural Transformations

**DOI:** 10.3390/nano13010192

**Published:** 2023-01-01

**Authors:** Sergey Kudryashov, Pavel Danilov, Nikita Smirnov, George Krasin, Roman Khmelnitskii, Oleg Kovalchuk, Galina Kriulina, Victor Martovitskiy, Vasily Lednev, Pavel Sdvizhenskii, Yulia Gulina, Elena Rimskaya, Evgeny Kuzmin, Jiajun Chen, Michael Kovalev, Alexey Levchenko

**Affiliations:** 1 Lebedev Physical Institute, 119991 Moscow, Russia; 2 Geo-Scientific Research Enterprise Public Joint Stock Company «ALROSA», 678175 Mirny, Russia; 3 Geology Faculty, Lomonosov Moscow State University, 119899 Moscow, Russia; 4 Prokhorov General Physics Institute, 119991 Moscow, Russia

**Keywords:** diamonds, ultrashort-laser pulses, filamentation, photoexcitation, transient stresses, high temperature, nanoscale impurity defects, atomistic structural transformations, optical impurity centers, photoluminescent encoding

## Abstract

The ultrashort-laser photoexcitation and structural modification of buried atomistic optical impurity centers in crystalline diamonds are the key enabling processes in the fabrication of ultrasensitive robust spectroscopic probes of electrical, magnetic, stress, temperature fields, and single-photon nanophotonic devices, as well as in “stealth” luminescent nano/microscale encoding in natural diamonds for their commercial tracing. Despite recent remarkable advances in ultrashort-laser predetermined generation of primitive optical centers in diamonds even on the single-center level, the underlying multi-scale basic processes, rather similar to other semiconductors and dielectrics, are almost uncovered due to the multitude of the involved multi-scale ultrafast and spatially inhomogeneous optical, electronic, thermal, and structural elementary events. We enlighten non-linear wavelength-, polarization-, intensity-, pulsewidth-, and focusing-dependent photoexcitation and energy deposition mechanisms in diamonds, coupled to the propagation of ultrashort laser pulses and ultrafast off-focus energy transport by electron–hole plasma, transient plasma- and hot-phonon-induced stress generation and the resulting variety of diverse structural atomistic modifications in the diamond lattice. Our findings pave the way for new forthcoming groundbreaking experiments and comprehensive enlightening two-temperature and/or atomistic modeling both in diamonds and other semiconductor/dielectric materials, as well as innovative technological breakthroughs in the field of single-photon source fabrication and “stealth” luminescent nano/microencoding in bulk diamonds for their commercial tracing.

## 1. Introduction

During the last decade, molecule-like optically active doping defects (optical centers, OC) in diamonds, related to nitrogen, boron, silicon, germanium, and phosphorus impurity atoms or clusters [1,2,3,4,5] (for a broader review see [6]), due to their fine spectral structure and mechanically robust, buried character demonstrated unprecedented spectral sensitivity in the nanoscale probing of electromagnetic, stress, and temperature fields [1,2,3,4,5,6,7,8,9,10]. Besides the macroscopic probabilistic approaches in fabricating OCs in diamonds, even at the single-photon source level, through the e-beam structural modification of a crystal [11] or the direct implanting of impurity atoms during CVD diamond growth [1,2,3,4,5], in the last few years, ultrashort pulse lasers were harnessed for the direct OC writing of arbitrary symbolic information at flexibly tunable pre-determined depths in such crystals [12,13,14]. This OC-based concept followed another one, exploiting OCs as active optical emitters to replace passive embedded graphitic microstructures [15,16,17,18] in bulk diamond micromarking/encoding [19,20].

Comparing to common free-running and Q-switched short pulse (nanosecond and longer) lasers, such direct ultrashort pulse (femtosecond, fs) laser writing appeared as a key enabling modality for low-energy and (sub)microscale well-controlled, deterministic nano-to-microscale engineering of the diamond lattice with its impurities at the atomistic structural and optical-range energy-spectrum levels [12,13,14,19,20,21,22,23]. However, despite these remarkable achievements in fs laser fabrication [12,13,14], the non-linear spectroscopy [24,25] and nanoscale mapping [26] of OCs in bulk diamonds, transient fundamental photogeneration and spatiotemporal relaxation processes for highly electronically excited states (local electron-hole plasma (EHP) density~10^21^–10^22^ cm^–3^) into the final incoherent atomistic structural modifications are almost completely uncovered [27,28]. Basically, this is still an evergreen problem of ultrafast non-thermal disordering in semiconductors and dielectrics [29,30,31,32], which microscopic physical picture was so far fragmentary, thus hindering the technological progress in the exploration of novel, prospective OC fabrication regimes. The common state of art for fs laser bulk damage in semiconductors and dielectrics, as well as for surface ablation, is missing the corresponding basic knowledge and related key parameters for a number of fundamental processes, governing ultrafast photoexcitation and relaxation [33,34,35].

First, in bulk transparent dielectric materials, high-power ultrashort laser pulses could propagate non-linearly, with polarization-, wavelength-, and pulsewidth-dependent χ_ʒ_-type Kerr self-focusing in the pre-focal region [36], as illustrated below in Section 2. Higher-order non-linearities χ_n_ in bulk dielectrics, responsible, e.g., for multi-photon EHP generation, could be also polarization-, wavelength-, and pulsewidth-dependent [36,37,38], while many dielectrics are yet unexplored. Furthermore, in natural diamonds with high OC concentrations (~10–10^3^ ppm), the strong enhancement of optical non-linearities could be expected due to presence of intra-gap intermediate states, positively adding to the high linear refractive index of the material (≈2.4) during self-focusing and filamentation against the negative EHP contribution, but key self-focusing parameters in diamonds are OC concentration-dependent and thus very controversial.

**Figure 1:**
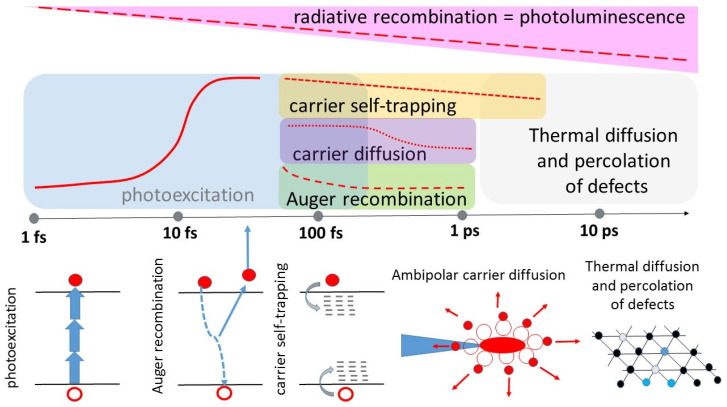
Timescales of consequent laser-diamond interaction and electronic/lattice relaxation processes upon intense ultrashort pulse laser photoexcitation. Red curves in the upper part illustrate temporal dynamics of electron–hole plasma (carrier) density, while the bottom part schematically exhibits basic principles of the involved relaxation phenomena under study.

Second, once the ultrafast non-linear photoexcitation of wide-band diamond started via multi-photon and avalanche ionization, its counteracting non-linear three-body Auger recombination process, which almost terminates the intensity-dependent growth of EHP density in the range~10^21^–10^22^ cm^–3^ in the fs-laser damage/ablation regime (Figure 1), is not characterized for the most part of semimetals, semiconductors, and dielectrics, as corresponding coefficients usually are not known. The reason is the photo-generated EHP density is usually probed in non-destructive photoconduction regimes at much lower densities (~10^14^–10^18^ cm^–3^) and other relaxation mechanisms, such as radiative recombination, surface recombination, trapping, etc., strongly predominate, making minor the Auger recombination contribution hardly distinguishable. Furthermore, the Auger recombination is a feeding electron, hole–phonon relaxation through the generation of hot carriers, with both these processes resulting in EHP-lattice energy transfer, which will be characterized in this study too.

Third, on surfaces and in bulk materials dense EHP rapidly diffuses out of the focal region in an ambipolar mode, as illustrated in Figure 1, with mobile electrons preceding the heavier, less mobile holes [39,40]. The corresponding diffusion coefficients were suggested to almost exponentially increase versus EHP density > 10^21^ cm^–3^ due to increasing plasma degeneracy [40], approaching, e.g., amorphous and crystalline silicon~10^3^–10^4^ cm^2^/s at EHP densities~10^21^–10^22^ cm^–3^ [41,42], and bringing the non-negligible picosecond EHP/energy transport around the focal region to a micro-scale. This makes the prompt EHP photogeneration dependent on high-NA focusing, regulates the prompt EHP absorption at high fs-laser intensities, when it predominates over the multi-photon intensity [43,44,45,46] and dictates the EHP relaxation timescale, as well as the Auger recombination.

Moreover, it prompts electronic and transient lattice-driven bandgap renormalization (up to 50% in the former case [47] and complete shrinkage overall [48]), becoming a crucial effect at EHP densities~10^21^–10^22^ cm^–3^ and could affect both EHP photogeneration [49] and the subsequent transport [50], recombination [51], and other relaxation dynamics. Such prompt bandgap renormalization effects, strongly modulating a spectrum of dielectric function of semimetals and semiconductors, could bring previously unobserved strong optical nonlinearities to the interactions of bulk diamonds with intense ultrashort laser pulses.

Finally, fifth, even though for the last two decades there were a few series of structural atomistic modeling in semimetals (graphite [52]), semiconductors (GaAs [53,54], silicon [55,56]), and dielectrics (diamond [27,28,57,58]) under intense fs-laser photoexcitation, the actual EHP-renormalized potential energy surfaces for EHP-driven (sub)picosecond atomic re-arrangements through displacive phase transitions or bulk catastrophic damage (Figure 1) are still not reliably clarified. Specifically, like a few other classes of materials [59], at the atomic level diamonds could generally undergo Frenkel-type damage with the simultaneous appearance of interstitial-vacancy (I–V) pairs, with the chemically labile neutral and negatively charged vacancies emerging separately as OCs in photoluminescence spectra [60] and joining the nitrogen interstitial to make a luminous neutral or negatively charged NV center [12,13,14,61]. Importantly, both carbon vacancies and interstitials could join different molecule-like OCs with their sets of electronic and vibrational states in the diamond bandgap [60,61]. In the context of intense ultrafast laser photoexcitation, the micro-scale “cloud” of I–V pairs locally photo-generated in the focal region, could dramatically reconfigure the former abundance of OCs, if any, in favor of the larger average fraction of bound vacancies. Such fs-laser-driven photochemical transformation was studied previously only in synthetic diamonds with their nitrogen impurity in the primitive atomic form, resulting in NV centers as single-photon emitters [12,13,14].

Overall, all the above-mentioned optical, electronic, and structural aspects of fs-laser–diamond interaction are not consistently explored up to the date, demanding the comprehensive spatial and temporal multi-scale characterization of diverse excitation and relaxation processes in different types of natural and synthetic diamonds with variable sets of initial nitrogen-based optical centers.

## 2. Basic Physical Processes of Ultrashort Pulse Laser-Diamond Interaction

### 2.1. Multi-Spectral Characterization of Native Optical Centers

**Figure 2:**
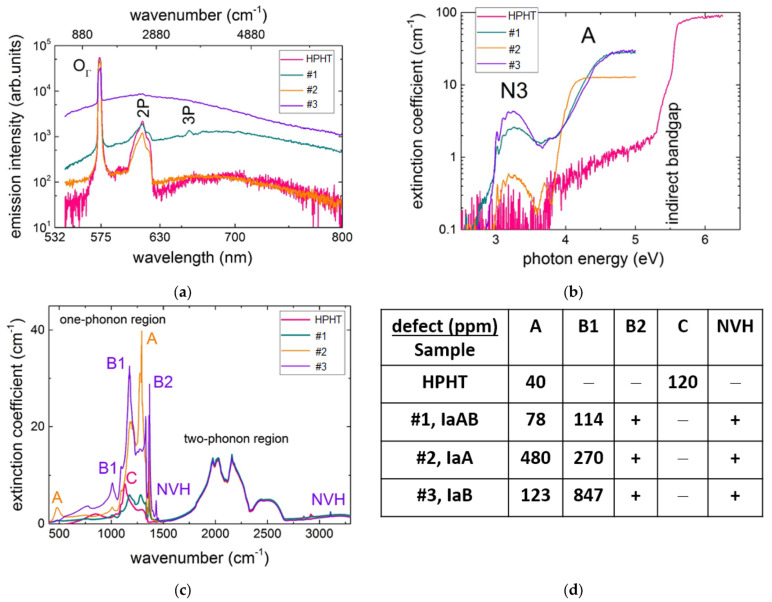
Characteristic spectra of diamonds IaAB, IaA, and IaB (#1–#3): (**a**) PL spectra at 532 nm excitation wavelength (spectral assignment after [60]); (**b**) UV-NIR optical density spectra, revealing main intra-gap absorption features (spectral assignment after [60]); (**c**) FT-IR absorbance spectra (spectral assignment after [60]); (**d**) abundance of different nitrogen-based defects (optical centers), according to FT-IR analysis.

Due to its high broadband transparency (direct bandgap–6.5 eV [62]), the multi-hundred plethora of native OCs emerges in the electronic and vibrational spectra of diamonds, being related to different rich chemical impurities [60,63,64]. As a result, their wavelength-dependent photoluminescence (PL) yield across the diamond bandgap, strongly varying in spectral and intensity terms, enables the ultrasensitive detection of characteristic Stokes emission bands of various PL-active OCs, usually red-shifted by 200–300 nm [60,65], and allows their routine gemological differentiation (Figure 2a). Meanwhile, according to the Stokes principle, luminous optically active centers can be identified by broadband UV-near-IR transmission spectroscopy [60] (Figure 2b), which reflects the homogeneous fundamental direct/indirect interband absorption and anti-Stokes OC absorption bands, involving intra-gap impurity states. In contrast, the structural spectral studies of OCs in the center-symmetric diamond lattice are usually related to IR-active but PL-blind “impurity atom-carbon atom” dipolar modes [60,64] and second-order intrinsic vibrational modes [60] (Figure 2c), while in the visible range, spatially resolved fs-laser second-harmonic generation was realized in a bulk diamond on unspecified (apparently, NV-type) dipolar optical centers [26]. Since the vast majority of OCs, besides free-excitonic states and A-band recombination traps [66,67], possess quasi-molecular energetic structure with ground and excited electronic states, equipped by the corresponding sets of vibrational levels [60,68], large displacements along their configuration coordinate or dampen radiative relaxation due to interstate conversion, which could result in negligible PL yield [69], requiring other characterization methods, such as electron paramagnetic resonance, to be invoked [70]. Center-symmetrical OCs allow their Raman detection via the excitation of coherent optical phonons [71]. On the other hand, non-center-symmetrical OCs could affect local second-harmonic generation inside diamonds, enabling their overall mapping [72].

Specifically, one HPHT and a few rather different IaAB, IaA, and IaB (#1–#3) natural diamonds with variable—low-to high (>40–1000 ppm)—nitrogen (N) content, utilized in this study, were semi-quantitatively evaluated regarding abundance of different centers by UV-vis (luminous substitutional 3NV/N3 and 2N/A centers [60], Figure 2b) and FT–IR (non-luminous substitutional 2N (A), 4NV (B1), N_∞_ (B2), N (C), and NVH centers, Figure 2c) transmittance in terms of their extinction coefficients. The evaluated relative abundance of different optically blind nitrogen-based centers is presented in the table in Figure 2d. Quite expectedly, stationary PL yield at 532 nm excitation exhibits strongly varying intensities for these diamond samples, with the maximal values for the diamond samples IaAB and IaB (Figure 2a, #1,#3), also demonstrating the maximal extinction (≈ absorption) coefficients for the highly aggregated nitrogen center 3NV (N3) and 2N (A) center in the spectral range of 3–5 eV (Figure 2b). In contrast, the sample IaA (#2), containing about 480 ppm of A centers (2N, 1282 cm^–1^) and 270 ppm of B1 centers (4NV, 1175 cm^–1^) and other non-polar centers, appears optically blind both in the PL and UV-vis spectra. A very similar optical response is demonstrated by the weakly luminescent HPHT diamond, containing mostly single-atom substitutional nitrogen impurity C centers (120 ppm, 1130 cm^–1^) and 40 ppm of A centers (Figure 2).

This brief overview illustrates, first, that despite the high overall nitrogen content, native substitutional nitrogen-based optical centers in synthetic and natural diamonds could appear in different chemical configurations, being absent or emerging in absorption/PL emission in different spectral ranges and requiring comprehensive UV-vis and IR spectral characterization. Second, even non-luminescent diamonds with rather high nitrogen content could be turned luminescent upon physico-chemical structural modification (aggregation, dissociation) of the present nitrogen impurities [61]. Third, optically active centers, such as intermediate intra-gap resonances, could contribute to optical non-linearities, affecting the ultrashort pulse laser photoexcitation of diamonds at the laser energy delivery and absorption/deposition stages during their nano- and micro-modifications.

### 2.2. Delivery of Ultrashort Laser Pulses in Bulk Diamonds: Linear Focusing versus Non-Linear Self-Focusing and Filamentation

Despite high impurity contents (up to 10^3^ ppm), resulting even in a rainbow of visible colorations (usually UV-range absorption for heavily nitrogen-doped colorless or yellow/brown diamonds) [61], where diamonds usually keep their high vis-NIR transparency. Such transparency implies the non-linear propagation of intense ultrashort laser pulses during their high-NA focusing inside diamonds, perturbing the anticipated linear (geometrical) focusing characteristics of direct writing regimes. High-order non-linearities are known to be related to multi-photon absorption (imaginary parts) and the generation of higher harmonics or wave-mixing (real parts), while low-order non-linearities emerge as laser beam Kerr self-focusing and filamentation (upstream non-linear focus), once the peak pulse power exceeds the critical one [36]. Critical powers for self-focusing on non-linear media are known to be wavelength-, polarization-, and even beam shape-dependent [36,37,38], while in diamonds the intra-gap OCs could function as intermediate resonances, enhancing such optical non-linearities [72].

**Figure 3:**
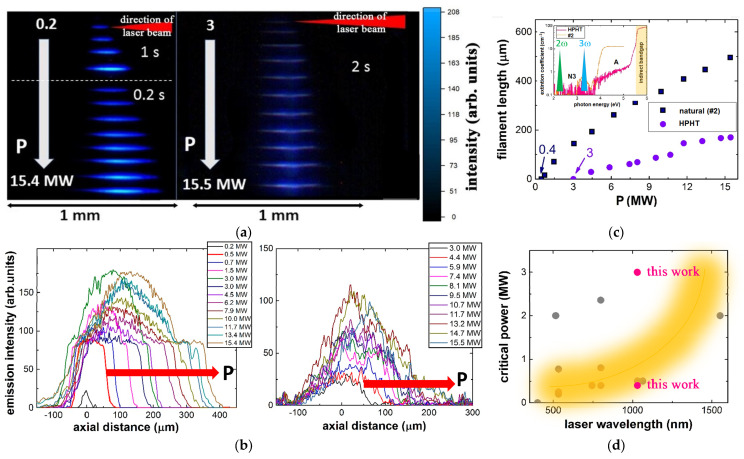
(**a**) Side-view images and (**b**) longitudinal profiles of PL luminous tracks acquired for 0.2–2 s inside natural (#2,a) and HPHT diamonds, which were excited by 300 fs and 1030 nm laser pulses focused by 0.25-NA objective at the different peak laser powers, demonstrating the upstream elongation in the filamentation regime; (**c**) Comparative dependences of filament lengths versus peak 1030 nm, 300 fs pulse power for the diamonds. Inset: spectra extinction coefficients of the diamonds and the corresponding second- and third-harmonic positions for the 1030 nm radiation (magnified view in Figure 2b); (**d**) Overview of our and previously measured critical power values *P_cr_* for diamonds in vis-NIR spectral range (data after [15,38,74,75,76,77,78,79]).

In our study, fs-laser microfilaments were side-view visualized as transient elongated luminous PL tracks during 0.25-NA focusing of 300 fs and 1030 nm laser pulses into the natural (#2, low concentration of optically active centers) and pure HPHT diamonds in the direct writing regime (Figure 3a) (1030 nm: peak power –0.2–15.5 MW). Initially, at the increasing peak laser power the linear focus was spatially symmetrically extended both up- and down-stream (Figure 3a–c) within the Rayleigh length scaled up by the high refractive index of diamond *n*_0_(1030 nm) ≈ 2.4 [73]

(1) $$L_{R}\left(\lambda ,NA\right)=\frac{\lambda }{n_{0}\left(\lambda \right)}\frac{n_{0}^{2}\left(\lambda \right)-NA^{2}}{\pi NA^{2}},$$

and additionally, by the spherical aberration, emerging at the air/diamond interface. Meanwhile, at higher peak laser powers >0.4 MW in the natural diamond and >3 MW in the HPHT diamond the longitudinal extension symmetry breaks, indicating the predominating extension of the PL tracks upstream (toward the pump fs-laser). Usually, if screening plasma shadow does not appear within the focal region in a bulk dielectric, such asymmetry could be considered as a solid indication of self-focusing and formation of non-linear focus prior to the geometrical (linear) one [36,38,74]. As a result, the critical power values *P_cr_* ≈ 0.4–0.5 MW for the natural diamond and ≈3 MW for the HPHT diamond can be derived at the 1030 nm wavelength [38,74] (Figure 3b) and compared to the previous measurements at the same and other vis-NIR wavelengths (Figure 3d).

The rising spectral trend for *P_cr_*(λ) in Figure 3d follows the well-known relationship for the Kerr self-focusing collapse arrested by the diffraction divergence of a short pulse laser beam [36,80,81]

(2) $$P_{cr}\left(\lambda \right)=\frac{3.77\lambda^{2}}{8\pi n_{2}\left(\lambda \right)n_{0}\left(\lambda \right)},$$

where, commonly, the linear refractive index *n*_0_(λ) ≈ const in the normal dispersion spectral range, while Kerr non-linear refraction coefficient *n*_2_(λ) is rapidly increasing toward shorter wavelengths [82]. In different colored and colorless diamonds with their different densities of intragap OC states as intermediate resonances, different cascaded contributions to χ^(3)^~*n*_2_(λ) are expected through corresponding wavelength-dependent linear optical susceptibilities κ^(1)^ in the form [72] of

(3) $$\chi^{\left(3\right)}\left(\lambda /3;\lambda ,\lambda ,\lambda \right)\propto \kappa^{\left(1\right)}\left(\lambda /3\right)\kappa^{\left(1\right)}\left(\lambda /2\right)(\kappa^{\left(1\right)}{\left(\lambda \right)}^{3}).$$

Here, the pronounced cascaded effect of κ^(1)^(λ/3) becomes evident due to the order of magnitude higher intragap N3-OC absorption in the natural diamond #2 in Figure 3c, inset (similarly, in Figure 2b), compared to the HPHT sample. This effect may also clarify the large dispersion range of critical power values (0.04–2 MW) in the literature [15,38,74,75,76,77,78,79].

However, for ultrashort laser pulses, the arrest of self-focusing, preceding the filamentation onset, is usually related to prompt plasma defocusing [36]

(4) $$n_{2}I\left(t\right)\approx \frac{\rho \left(t\right)}{2\rho_{cr}},$$

where *I(t)* is the instantaneous laser intensity, *ρ(t)* is the electron–hole plasma density, tending to the critical plasma density *ρ*_cr_ ∝ λ^2^. For comparison, group velocity dispersion [83] also plays minor role for the tightly focused ultrashort laser pulses, while other possible arresting factors—the saturation of n_2_ [84], non-linear losses, vector, and non-paraxial effects [36]—appear not yet be justified. As a result, the common concept of critical pulse power for the onset of filamentation in Equation (2) becomes invalid for ultrashort laser pulses, as Kerr self-focusing is arrested not by diffraction but by plasma defocusing or other abovementioned factors [36,83,84]. Hence, other optical invariants are required to characterize the delicate balance between self-focusing and potential defocusing factors.

**Figure 4:**
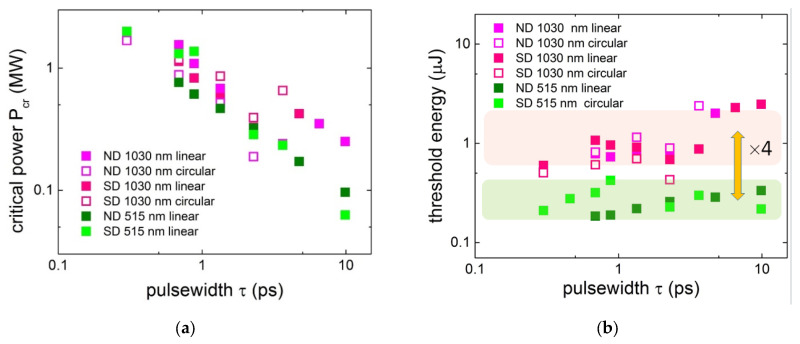
Critical peak pulse power *P_cr_* (**a**) and threshold pulse energy *E_th_* (**b**) for the onset of filamentation in natural and synthetic diamonds at 515 nm and 1030 nm wavelengths versus laser pulsewidth (adapted from [38]).

In this context, recent experiments [38] demonstrated that during the propagation of positively chirped ultrashort laser pulses of variable (0.3–12 ps) pulsewidths in non-linear dielectric media, such as diamonds, the critical power *P_cr_* ceases to be an invariant, as in short pulse experiments, where the pulsewidth-independent beam divergence prevents the Kerr self-focusing beam collapse [36,80,81]. In contrast, in the ultrashort pulse laser experiments, the threshold parameter for filamentation onset appears to be the pulse energy *E_th_*, once the threshold power *P_cr_* for the filamentation onset exhibits the reciprocal dependence [38] (Figure 4a). This finding apparently reflects the fact that the temporally increasing lattice polarization by the laser pulses enhances the prompt Kerr nonlinearity via a time-delayed Raman–Kerr effect [36], thus requiring lower peak pulse intensity/power to balance the same near-critical plasma defocusing. Again, the stronger nitrogen impurity-doped natural diamonds systematically demonstrate the lower threshold energy for filamentation, compared to the purer synthetic ones (Figure 4b). Moreover, the two-fold shorter wavelength—515 nm versus 1030 nm—corresponds to the four-fold lower threshold energy (*E_th_* ∝ λ^2^), in the agreement with Formula (4). This implies that the arrest of self-focusing collapse occurs by sub-critical electron–hole plasma independently relative to the (sub)picosecond laser pulsewidth, while the local laser intensity in the collapse region is high enough to produce such plasma (see Section 2.3 below). This is also strongly supported by the similar magnitudes of *P_cr_* and *E_th_* for the linear and circular laser polarizations, indicating that plasma absorption, rather than nonlinear photoionization dominates at the filamentation onset.

### 2.3. Pulsewidth- and Polarization-Dependent Ultrafast Laser Photoexcitation

**Figure 5:**
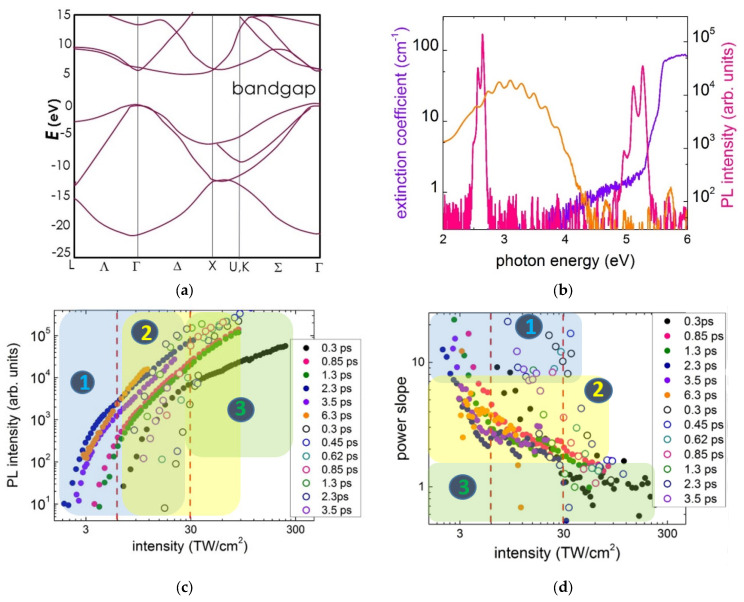
(**a**) Band spectrum of diamond. (**b**) Typical PL spectra of A-band and FE-recombination bands in the natural (#1) and HPHT diamonds, respectively (spectral assignment after [60,64,65]); (**c**) PL yield dependences for the diamonds at 1030 nm laser intensity at different pulsewidths and (**d**) their corresponding non-linearity slopes *N_ϕ_*. Dark and light dots represent HPHT and natural diamonds, respectively. The highlighted regions and numbered circles denote the corresponding EHP dynamics regimes #1–#3. Adapted from [91].

Recently, dynamic photoluminescence micro-spectroscopy emerged as an ultrasensitive and informative tool in acquiring spectral information and photophysical dynamics of intra-gap OC states excited by near-IR ultrashort laser pulses [85,86,87], and broad spectra of intra-gap OC states excited by visible-range ultrashort laser pulses [88]. Meanwhile, PL spectroscopy is also known to provide insights into interband photoexcitation processes (Figure 5a), probing low-density free-exciton gas and low-density (~10^15^–10^17^ particles/cm^3^) electron–hole plasma liquid dynamics at later stages of their radiative relaxation [89,90]. However, for the current development of ultrashort pulse laser writing technology in diamonds it is required to envision intensity- and wavelength-dependent photoexcitation mechanisms and electronic dynamics in dense electron–hole plasma, similar to that explored in other dielectrics by time-resolved interferometry [43,44,45] and ultrasonics [46]. In this context, despite the radiative recombination and the related PL being a rather slow and low-rate process during the photoexcitation of diamonds at high fs-laser intensities (Figure 1), it effectively functions as a non-perturbing indicator of EHP density, produced via different photogeneration and recombination processes [91]. Specifically, UV-range (>350 nm) A-band emission, resulting from radiative recombination of electrons and hopes trapped by donor–acceptor pairs in natural diamonds [67,87,91], and deep UV (>230 nm) bands originating from predominating free-exciton radiative recombination in synthetic diamonds [66,89,90] (Figure 5b), are known to provide the most straightforward insight into the preceding dense EHP dynamics.

The peak intensity dependences of PL yield were obtained for the A-band in the natural diamond (#1) and main free-exciton recombination band in the HPHT diamond (Figure 5a,b) photoexcited by 0.25-NA focused 1030 nm ultrashort laser pulses of different pulsewidths (0.3–6.2 ps). These dependences exhibit the non-linear trends at lower intensities, typical for multi-photon absorption [43,44,45,46] and saturation, or nearly linear trends at higher intensities, usually related to the formation of opaque electron–hole plasma or filamentation [46,88] (Figure 5c,d), where the non-linearity parameter (power slope) *N*(*I*_0_) can be derived from the continuous PL yield dependences on the peak intensity *I*_0_ as follows:

(5) $$N_{\Phi }\left(I_{0}\right)=\left(\frac{d\Phi }{dI_{0}}\right)\frac{I_{0}}{\Phi }.$$

The continuous variation of *N_ϕ_*(*I*_0_), starting in Figure 5d from the magnitudes > 10 (well above than in the previous studies [89,90]) and lasting until ≈ 1, for the first time indicate the gradually and continuously changing intensity-dependent roles/contributions of different contributions in the PL-tracked inter-band photoexcitation and accompanying free-carrier/exciton dynamics. Importantly, comparing to other EHP characterization methods (interferometry [43,44,45], ultrasonics [46]), PL microspectroscopy tracks not the density of separate carriers but the interaction, i.e., radiative recombination, of their pairs, exhibiting case-specific non-linearities, illustrated below in the framework of EHP-related kinetic rate model. Moreover, utilizing the results of our measurements of the critical powers for self-focusing at the 1030 nm wavelength in the natural (0.5 ± 0.4 MW [38,74,88], *I*_0_ ≈ 5 TW/cm^2^) and HPHT (3 ± 1 MW, *I*_0_ ≈ 30 TW/cm^2^) diamonds, one can rule out the high-intensity linear range of these dependences as related to the (multi)filamentary propagation regime [46,92], where the laser intensity is not well-defined, even though it is clamped in each separate filament [36].

Recently, for such an analysis of EHP and PL dynamics, a kinetic rate model was enlighteningly used in the common form [93], relating multiphoton and impact ionization, Auger and radiative recombination as the consecutive terms, respectively, and describing the corresponding PL yield as follows:

(6) $$\frac{d\rho_{eh}}{dt}=\sigma_{N}I^{N}+\alpha I\rho_{eh}-\gamma \rho_{eh}^{3}-\beta \rho_{eh}^{2},\;\;\Phi \propto \int \beta \rho_{eh}^{2}dt.$$

At lower laser intensities of *I*_0_~1 TW/cm^2^, one can expect the multi-photon seeding of the ultralow density gas of electron–hole pairs (free excitons) via direct photoexcitation across the direct interband bandgap, ranging from the minimal value E_dir_(Γ) ≈ 6.5 eV to the maximum one ≈12 eV at the X, U, and K points of the Brillouin zone [62] (regime #1). The ponderomotive extension of the bandgap [94,95] and the splitting of the triply degenerate valence bands by the linearly polarized laser field [95] are minor (<1 eV) in the intensity range. This means that, depending on the orientation of the laser polarization regarding the crystallographic axes [62], the number of the necessary 1030 nm laser photons (photon energy ħω ≈ 1.2 eV) varies from N = E_dir_/ħω ≈ 6 (Γ-point) to 10–11 (X, U, and K points). At the ultralow density of the electron–hole pair gas, each electron–hole pair or resulting free exciton recombine separately in the concerted way and the power slope for the PL yield is expected to be equal to N (see, e.g., five-photon PL yield for 780 nm fs-laser photons in [89]). However, if the photo-generated electron-hole pairs become intermixed in the EHP fluid, they lose their correlation established during the photogeneration and appear independently in the excitonic recombination with the 2N-fold slope (the low intensity bluish region in Figure 5c,d).

(7) $$\frac{d\rho_{eh}}{dt}=\sigma_{N}I^{N},\rho_{eh}\propto I_{0}^{N},\Phi \propto \int \rho_{eh}^{2}dt\propto I_{0}^{2N}.$$

Specifically, in our experiments, the maximal observed *N_ϕ_* values < 20 are reasonably consistent with the 2N = 12–22 expected in this irradiation regime for the arbitrary orientation of the linear laser polarization regarding the crystalline axes in diamond.

At the slightly higher, medium laser intensities *I*_0_~3 TW/cm^2^ and EHP densities *ρ_eh_*~10^21^ cm^–3^, three-body Auger recombination turns on in the regime #2, limiting the photoexcited EHP density and providing its ultrafast relaxation

(8) $$\frac{d\rho_{eh}}{dt}=\sigma_{N}I^{N}-\gamma \rho_{eh}^{3}-\beta \rho_{eh}^{2},\sigma_{N}I^{N}\approx \gamma \rho_{eh}^{3},\rho_{eh}\propto I_{0}^{N/3},\Phi \propto \int \rho_{eh}^{2}dt\propto I_{0}^{\frac{2N}{3}},$$

reducing the PL-yield non-linearity slope down to the predicted values 2N/3 = 4–7, which are in good agreement with our experimental observations for *N_ϕ_* (the medium intensity yellowish region in Figure 5d). The laser-induced prompt ponderomotive extension of the bandgap and the splitting of the triply degenerate valance bands by the linearly polarized laser field are still minor (<1 eV) in this intensity range. As a result, some kinks at *N_ϕ_*~4–6 were observed on the dependence *N_ϕ_*(*I*_0_) for the HPHT diamond in Figure 5d, being consistent with the expected values 2N/3 = 4–7.

Finally, at high laser intensities *I*_0_ > 10 TW/cm^2^ and near- or critical EHP density (*ρ_eh_*~10^21^–10^22^ cm^–3^), the plasma becomes balanced through the reverse impact ionization and Auger recombination processes in regime #3:

(9) $$\frac{d\rho_{eh}}{dt}=\alpha I\rho_{eh}-\gamma \rho_{eh}^{3}-\beta \rho_{eh}^{2},\alpha I\rho_{eh}\approx \gamma \rho_{eh}^{3},\;\;\rho_{eh}\propto \sqrt{I_{0}},\Phi \propto \int \rho_{eh}^{2}dt\propto I_{0},$$

resulting in the linear PL yield, which is also observed in our study for the HPHT diamond at higher intensities (the high intensity greenish region in Figure 5c,d). In this regime, the laser intensity is high enough to induce a strong ponderomotive effect and the laser-field splitting of the degenerate valence bands; however, femtosecond-scale carrier collisions in dense EHP could strongly dampen these effects. In contrast, prompt EHP-induced bandgap renormalization could occur during the ultrashort pump laser pulses alike to other insulators [47,48,49]. However, both the laser-field and EHP-driven bandgap modulations do not appear explicitly in *N_ϕ_*, even though they could strongly modulate both the impact ionization and Auger recombination rates in Equation (9). Furthermore, the formation of dense, near-critical EHP was demonstrated to support the onset of filamentation of ultrashort laser pulses in condensed media by counter-balancing the Kerr self-focusing effect [36,46,92].

**Figure 6:**
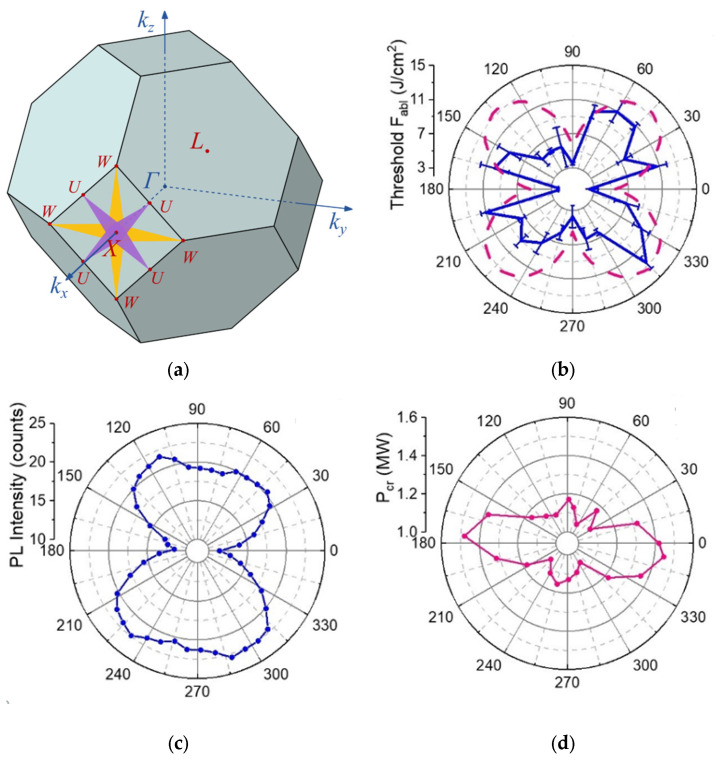
(**a**) Brillouin zone of a diamond and its main directions; (**b**) Polar plot of single-shot 1030 nm, 300 fs-laser ablation thresholds for the 100-facet diamond surface as a function of polarization angle; The pink dashed pattern denotes the direct bandgap variation in diamond; (**c**) Polar plot of bulk PL yield collected in natural diamond in the [110] direction and excited by linearly polarized 1030 nm and 300 fs-laser pulses focused inside it along the 110 axis; (**d**) polar plot of the threshold energy of filamentation onset in natural diamond for linearly polarized 1030 nm and 300 fs-laser pulse focused inside it along the [110] axis. Adapted from [37].

Furthermore, despite the high cubic symmetry of diamond, its non-linear optical interactions with polarized ultrashort laser pulses appear polarization-sensitive in the diverse directions of its Brillouin zone (Figure 6a). Specifically, polarization effects in the the nonlinear photoexcitation of diamond by linearly polarized 1030 nm laser pulses of variable pulse widths (0.3, 1, and 3 ps) were explored by studying single-shot surface ablation thresholds for the (100)-facet diamond plates (Figure 6b, the case of 0.3 ps pulses and 100-facets), multi-shot A-band PL yields (Figure 6c, 0.3 ps), and threshold energies for filamentation (Figure 6d, 0.3ps). The rotation of the laser polarization in the surface plane or in the bulk material resulted in the varying orientation of the laser field along the different crystallographic axes in diamond, promoting its direct photoexcitation in the corresponding U and W points of the Brillouin zone across the considerably different bandgaps of 11.4 and 18.1 eV [62] (Figure 5a,f), respectively. As a result, in the case of the laser polarization in U points, the single-shot ablation threshold varies about 4 J/cm^2^, while in W points it increases to 7–8 J/cm^2^ (Figure 6b), comparing to the weak effects in photogeneration rates previously observed for linearly polarized fs-laser pulses propagating in IIa-diamond along the [110] and [100] axes [90] (it is of note, longer picosecond laser pulses provided much lower difference in the ablation thresholds under photoexcitation in the U and W points (not shown)). Similarly, multi-photon PL yield collected in the [110] direction, exhibited two-fold symmetry of the polar plot (Figure 6c) for the linearly polarized fs-laser pulses of the even laser energies/intensities, propagating along the 110 axis, with the azimuthal polarization pattern reflecting the local symmetry in the transverse direction. Likewise, such azimuthal polarization patterns emerged in the polar plot for the threshold fs-laser pulse energies (Figure 6d), corresponding to the onset of micro-scale filamentation in the diamond along the [110] axis (see Figure 4 and its related discussion above).

Overall, our systematic studies of polarization effects in bulk ultrashort pulse laser photoexcitation in cubic diamond indicate that strongly varying thresholds could be expected for non-linear optical surface and bulk interactions in the material and its related ultrashort pulse laser patterning with the previously reported thresholds strongly differing for the missing control of laser polarizations and crystal orientations [96,97,98].

### 2.4. Ultrafast EHP Energy Transport and Transfer into Lattice

Ultrafast micro-scale energy deposition in semiconductors and dielectrics via the generation of dense EHP during the ultrashort laser pulses results in the accompanying spatial extension of the plasma and related deposited energy by means of ambipolar carrier diffusion [39]. Such EHP transport occurs in semiconductors and dielectrics along with the coupled three-body (eeh, ehh) Auger recombination and the relaxation of hot carriers in the lattice via the emission of optical phonons [99] (Figure 1), with their joint dynamics characterized by the timescale of the latter process; as a result, EHP density can be drastically reduced to 10^20^–10^21^ cm^–3^ [100], while exciting the atomic motion. The corresponding Auger recombination coefficients were accurately derived yet only for a limited number of popular semiconductors (Si [101], GaAs [102] etc.), while the related ambipolar plasma diffusion coefficients *D_a_*~10–102 cm^2^/s were evaluated only at moderate EHP densities~10^19^–10^20^ cm^–3^ in Si [39] and ~10^21^ cm^–3^ in diamond [103]. Meanwhile, at higher EHP densities the ~10^20^–10^22^ cm^–3^ ambipolar diffusion coefficients were predicted to increase almost exponentially to much higher magnitudes *D_a_* versus plasma density owing to its increasing degeneracy in the room temperature lattice [39,40]. Indeed, recently ambipolar diffusion coefficients as high as *D_a_*~104 cm^2^/s were reported in crystalline c-Si (*D_a_*~10^3^–10^4^ cm^2^/s in amorphous a-Si [41]) at high EHP densities~10^21^–10^22^ cm^–3^, driving the microscale ambipolar electron–hole plasma diffusion on picosecond EHP-lattice relaxation timescale [2]. Specifically, upon c-Si or a-Si surface photoexcitation by the laser pulses of variable pulsewidths (0.3–10 ps), such pulsewidth-dependent ultrafast lateral carrier/energy transport producing micrometer-broadened single-shot surface craters beyond the focal region for laser pulse durations shorter than the Auger recombination/EHP-lattice relaxation time *T_th_*~2 ps [41,42]. Hence, both important characteristics of EHP relaxation (Auger recombination and ambipolar diffusion transport) dynamics and energy transfer into lattice, which are usually unknown in semiconductors and dielectrics, were derived by the original method.

**Figure 7:**
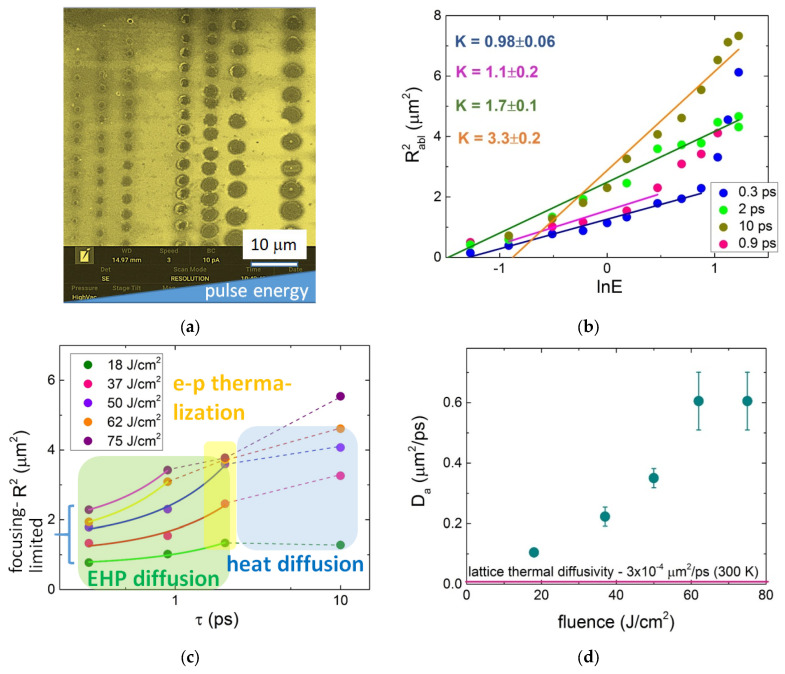
(**a**) Typical single-shot craters on natural diamond surface produced by 1030 nm laser pulses of variable pulsewidths τ = 0.3–10 ps and pulse energies, focused at NA = 0.65; (**b**) dependence R_abl_^2^lnE [µJ], showing the characteristic squared energy deposition radii as linear slopes K; (**c**) R_abl_^2^ dependences (color circles and dashed curves) on lgτ with linear fitting slopes for R_abl_^2^ τ (solid lines), which start at the focusing-limited squared radii at ambipolar diffusion timescale of 0–2 ps, ending up with electron–phonon (e–p) thermalization and heat diffusion; (**d**) derived ambipolar diffusion coefficient *D_a_* as a function of laser fluence and the horizontal line showing the corresponding maximal room temperature lattice thermal diffusivity χ ≈ 3 × 10^–4^ µm^2^/ps (3 cm^2^/s [104]).

In this study, similar single-shot ablation craters (Figure 7a) were produced on diamond surfaces under their irradiation by 1030 nm laser pulses of variable pulsewidths (0.3–10 ps), providing the dependences of the squared crater radii R_abl_^2^-lnE (Figure 7b). Starting from the focusing-limited minimal values at 0.3 ps, R_abl_^2^ systematically and significantly increased at the same pulse energies versus τ_las_ in the range of 0.3–2 ps and just a little or even saturate at τ_las_~2–10 ps (Figure 7c). This trend could indicate almost no ultrafast electronic transport for τ_las_ > 2 ps (≈2*T_th_*), since lattice transport at the room temperature thermal diffusivity coefficient χ ≈ 10 cm^2^/s rapidly decreases to χ~1 cm^2^/s at higher temperatures <1000 K [104], becoming negligible on the ps-timescale. Here, the observed change in energy transport length at ≈1–2 ps enables, for the first time, to relate this time window to the characteristic EHP-lattice relaxation time *T_th_* ≈ 1 ps in diamond, accounting for both Auger recombination, generating hot carriers, and the accompanying EHP-lattice transfer.

Hence, very similarly to Si, one can consider both considerable Auger recombination and carrier-lattice thermalization terminated at 2 ps. The corresponding diffusive transport coefficient was evaluated as follows:

(10) $$D_{a}\left(I_{0}\right)\approx \frac{R_{abl}^{2}\left(\tau ,I_{0}\right)-R_{abl}^{2}\left(0.3ps,I_{0}\right)}{\pi \tau },$$

fitting the pulsewidth-extended crater diameters at the shorter times < 2 ps (Figure 7c) regarding their minimal, focusing-limited values at τ_las_ = 0.3 ps (1/e-radius ≈ 1 µm according to Liu’s procedure [105]), when taken at the same incident pulse energies and focusing conditions. The resulting derived values *D_a_*~10^3^–10^4^ cm^2^/s almost exponentially increase versus laser fluence, which is scalable to EHP excitation level and exceed by orders of magnitude not only the maximal room temperature lattice thermal diffusivity value of χ ≈ 3 cm^2^/s [104] but also the previously measured ambipolar diffusion coefficient *D_a_*~10^2^ cm^2^/s [103].

The derived ambipolar EHP diffusion coefficient *D_a_*~10^3^–10^4^ cm^2^/s, as one of the main fitting parameters in plasma dynamics models, indicates the critical importance of the ultrafast EHP transport in diamond, rather neglected in the previous simulations [96,106,107,108]. This ultrafast micro-scale EHP process has an important implication in direct laser writing in diamonds, differently affecting prompt EHP and related energy density in the focal region upon laser focusing with different NA > 0.1 (the higher focusing NA and smaller the (sub)micron focal spot, and the stronger corresponding transport-based reduction). As mentioned above, altogether T_th_ and D_a_ provide a solid ground for EHP-density dependent two-temperature model [27,96,106,107,108] description of EHP and lattice temperature dynamics in diamond in the generalized form (as compared to the comprehensive model in [39]) for the EHP density conservation

(11) $$\frac{\partial \rho_{eh}}{\partial t}+\nabla \cdot \left(-D_{a}\nabla \rho_{eh}\right)=Q_{\rho },$$

dictated by the ambipolar diffusive plasma flux (left side, second term) and right-side EHP source term composed by photogeneration and Auger recombination/EHP-lattice relaxation contributions. The EHP density dynamics is coupled to its energy density dynamics [39]

(12) $$\frac{\partial U}{\partial t}+\nabla \cdot W=Q_{e}-C_{e}\frac{T_{e}-T_{l}}{T_{th}},$$

where *U* in the EHP internal energy, *W* is the electronic heat flux via ambipolar diffusion, *Q_e_* is the absorbed energy density, while *C_e_* and *T_e_*(*_l_*) are the EHP heat capacity and instantaneous EHP (lattice) temperature, respectively. Here, again EHP energy flux and EHP-lattice energy transfer are managed by *T_th_* and *D_a_* quantities. Finally, due to the much lower lattice thermal diffusivity χ ≈ 3 cm^2^/s in diamond [104] and the related thermal energy flux *q*_l_, it is that EHP-lattice relaxation (right-side term) dictates the mostly temporal evolution of lattice temperature *T_l_*

(13) $$\rho C_{P}\frac{\partial T_{l}}{\partial t}+\nabla \cdot q_{l}=C_{P}\frac{T_{e}-T_{l}}{T_{th}},$$

where *C_P_* is the lattice heat capacity. Thus, overall, the *T_th_* and *D_a_* quantities appear as the key parameters in the EHP-density dependent two-temperature model, paving the way toward the quantitative description of ultrafast electronic and lattice dynamics in diamonds.

### 2.5. Ultrafast Lattice Dynamics Envisioned by In Situ Raman Scattering

**Figure 8:**
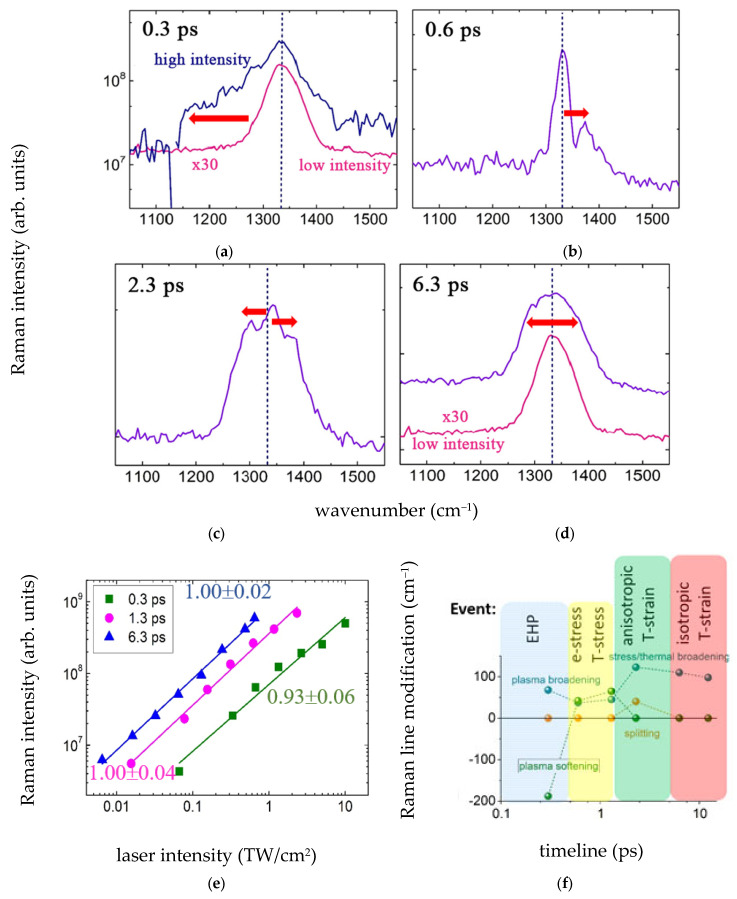
(**a**–**d**) Stokes’ first-order Raman spectra of the center-zone optical phonon in natural diamond #1 at variable 515 nm laser pulsewidths of 0.3, 0.6, 2.3, and 6.3 ps, respectively. The red horizontal arrows show broadening, shifting, and splitting effects. The low-intensity reference line corresponds to the 0.3 ps photoexcitation at 0.24 TW/cm^2^; (**e**) linear log–log dependences of Raman intensity on laser intensity at the different pulsewidths with their power slopes; (**f**) timeline of lattice relaxation processes emerging in the Raman spectra (**a**–**d**) according to their shifts, broadening and splitting. Adapted from [112].

Picosecond-scale ultrafast Auger recombination and electron–phonon relaxation in diamond anticipates the corresponding local pressure buildup in the focal volume, which was theoretically predicted to approach ~TPa magnitudes [109], relaxing for the one-micron wide focal spot through initial shock-wave propagation and later on a sub-nanosecond timescale w/*C*_l_~10^2^ ps at longitudinal sound velocity~10 km/s [104]. In this study, such high compressive stresses (high-pressure, HP), if any, could affect point defect/color center configurations and abundances in the focal region through the same thermodynamic and kinetic factors, which regulate their steady-state high-temperature (HT) structural transformations [61]. Meanwhile, the peculiarity of the ultrashort pulse laser irradiation of diamond is not only the generation of Frenkel “interstitial-vacancy” (I–V) pairs, but also non-equilibrium isotropic EHP-generated stresses [110,111], converting upon into anisotropic thermal ones during EHP lattice relaxation. Hence, the impulsive excitation of phonon modes can be tracked by spontaneous or stimulated Raman scattering, increasing the tracking timescale via laser pulse stretching by introducing a positive chirp. Below, in Figure 8, we present experimental results on spontaneous off-resonant Raman spectra of the center-zone optical phonon in natural diamond, while their different appearances at the variable pulsewidth demonstrates the temporal lattice dynamics as a function of EHP density and pressure or thermal pressure.

In these experiments, the 515 nm ultrashort laser pulses focused into the natural diamond #1 and the emission scattered at the normal angle was spectrally analyzed (with the 515 nm pump line blocked by a notch filter) in the range, corresponding to the first-order Raman line at 1332 cm^–1^ (Figure 8a–d). The Raman line intensity linearly increased versus laser intensity (Figure 8e), provided by the increasing pulse energy at the fixed focusing NA = 0.25, thus indicating the spontaneous off-resonant Raman scattering (the scattered spectral component is missing in the initial laser spectrum) instantaneously during the ultrashort laser pulses.

First, the ultrafast red-shifting (<190 cm^–1^) and broadening (≈70 cm^–1^) of the optical-phonon Raman line regarding its low-intensity, laser line-limited halfwidth ≈60 cm^–1^ (in single-mode CW Raman spectroscopy, ≈1.7 cm^–1^ [113]) can be observed under the diamond excitation by the 0.3 ps laser pulses (Figure 8a,f), indicating the instantaneous softening of the optical phonon during the pulses by the photogenerated EHP [49]

(14) $$\Delta \omega \left(\rho_{eh}\right)\approx 2\underset{\alpha \neq \beta ,k}{\sum }\frac{{\left|M_{\alpha \beta k}\right|}^{2}}{h\varepsilon_{\alpha \beta k}}\frac{n_{\alpha k}}{N_{0}}\approx C\rho_{eh},$$

where *M_αβk_* is the off-diagonal e,h-optical phonon coupling matrix element for the bands α and β separate by the transition energy *ε_αβk_*, *n_αk_* is the occupation number for the band α, *N*_0_ is the calibration constant, and *C* is the effective numerical factor. According to the anticipated magnitude of the optical deformation potential Dopt (ODP) in diamond *D*_opt_ ≈ 5 eV [103], the observed prompt excitation of the optical phonon (diagonal ODP elements) and its softening (unknown off-diagonal ODP elements) are quite possible. Based on the softening effect, which should give Δω ≈ ω_0_ at the 0.1*N*_V_ ≈ 6 × 10^22^ cm^–3^ [49] for the total density of valence electrons in diamond *N*_V_ ≈ 6 × 10^23^ cm^–3^, one can roughly evaluate the EHP density *ρ_eh_* as

(15) $$\rho_{eh}\approx \frac{N_{V}}{10}\frac{\Delta \omega }{\omega_{0}}\sim \left(3-7\right)\cdot 10^{21}\;cm^{–3},$$

which is consistent with the transient critical density ~10^21^ cm^–3^. It is worth noting that higher maximal optical phonon softening could occur for much higher EHP density at the end of the pump pulses.

Second, EHP interaction with the center-zone acoustic phonon is known to produce isotropic stress [111,114], which builds up in a quantum manner through consequent phonon emission in consequent delayed steps on a sub-picosecond timescale [115]. Specifically, in the diamond the transmitted 0.6 ps or 1.3 ps laser pulses, comparing to the 0.3 ps pulses, show the difference in the appearance of the second minor peak, blue-shifted by 40–65 cm^–1^ (Figure 8b,f). The peak shift increases at higher laser intensities and could be related to the trailing part of the slightly longer laser pulses. This blue shift of the Raman line can be related to homogeneous EHP-induced stress [110,111]

(16) $$\sigma_{el}\left(\rho_{eh}\right)\approx D_{ac}\rho_{eh},$$

which is σ_el~_15–20 GPa through the calibration relationships ∝ 3.2 cm^–1^/GPa [60,113], in agreement with the estimate σ*_el_*~*D_ac_ρ_eh_*~10 GPa for the acoustic deformation potential value *D_ac_* ≈ 10 eV [103] and *ρ*_eh~_10^22^ cm^–3^ and somewhat exceeding the predicted values in [116]. The isotropicity of the stress could result from the predominant initial EHP excitation of the center-zone acoustic phonon [114], similarly to the thermal pressure build-up. Alternatively, one can consider such a double-peak spectrum of the optical phonon as its splitting into unaffected doublet and shifted singlet due to EHP-induced compressive uniaxial stresses along the 111 or 100 axes [117], resulting in Δω[111] ≈ 2.2 cm^–1^/GPa and Δω[100] ≈ 0.73 cm^–1^/GPa [60,113], respectively. Such stresses could be generated by non-equilibrium (“hot”) long-wavelength acoustic phonons during hot-carrier relaxation in X-valleys (100 axis stress). Furthermore, 111-type splitting could result from a “silent” phonon excited as sub-lattice displacement along with the center-zone optical phonon [118].

Finally, for the larger laser pulse-widths of 2.3 ps and 6.3 ps (Figure 8c,f), one can observe triple symmetric splitting of the Raman line (two split singlets around the unbiased one), indicating the effect of even less symmetric, biaxial, or even tri-axial stresses. Such temporal transformation of the Raman line potentially exhibits anharmonic decay-driven phonon–phonon relaxation [119], resulting in other transient “hot” modes and related symmetry of stress fields, in agreement with the electron-lattice thermalization time ≤2 ps measured above (Figure 7). The observed splitting Δω~30–40 cm^–1^ could be associated, according to the relationship Δω ∝ (0.7–2.2 cm^–1^/GPa)σ [60,113], with the “thermal” stress magnitudes~20–60 GPa which are consistent with σ*_el_*~20–30 GPa. The non-equilibrium, “hot” phonon character of the transient anisotropic pressure is demonstrated in Figure 8d,f, where the longer 12.3 ps and even the high-intensity 6.3 ps laser pulse result again in the smooth, but strongly broadened (alike to the total triplet width in Figure 8c), Raman line, consistent with the final thermalization of the deposited laser energy. Accounting for the ultrahigh bulk modulus of diamond,~900 GPa [104], the revealed electronic and thermal stresses <60 GPa are obviously insufficient to drive strong shock waves and unload the stressed focal volume on the picosecond timescale.

Hence, the spontaneous optical–phonon Raman scattering, pumped and probed in this study by ultrashort chirped laser pulses of variable fs/ps widths, enlightens the timeline of phonon dynamics in photoexcited diamond from the sub-picosecond EHP-induced phonon softening/damping, through (sub)picosecond electronic stress blue-shifting, to anisotropic splitting by the picosecond hot phonon or thermal stresses (Figure 8f). These high transient stresses, emerging upon the ultrashort pulse laser excitation, could affect either the local concentration of point defects in the focal region or induce their structural transformations, similarly to static ones [61].

## 3. Novel Atomistic Paths in Ultrashort Pulse Laser Tailoring of Optical Nitrogen Centers in Diamonds: Making Visible Invisible and Back

Ultrafast laser photoexcitation through the accompanying electronic and lattice relaxation processes induces in the focal or filamentation volumes inside diamonds atomistic structural transformations, involving the photo-injection of labile Frenkel “interstitial-vacancy” pairs (“laser damage”) [88]. These photogenerated active intrinsic atomistic defects could renormalize the initial abundance of different OCs in diamonds, for example, by locally joining and reconfiguring in the focal volume the present atomic-like substitutional nitrogen (N) C centers in ultrapure (nitrogen content~1 ppb) electronic-grade synthetic diamonds to yield NV centers as single-photon sources [11,12,13,14,21,22,23]). In contrast, in a nitrogen-enriched (~100 ppm) red-colored synthetic diamond, the opposite occurs; a laser-induced vacancy-driven aggregation process of C and NV centers was recently demonstrated [120], appearing as its permanent visual bleaching.

Recently, similar final OCs were observed upon ultrafast laser photoexcitation in natural diamonds highly enriched by different medium-based and highly aggregated OCs, with the overall nitrogen concentration up to 10^3^ ppm [121]. Alternatively, direct intra-center photoexcitation [85,86,87,88,91,122,123] or indirect excitation via interband EHP photogeneration and succeeding (non)radiative intra-gap relaxation of electrons and holes could take place for different OC states in natural diamonds [123], potentially resulting in their (partial) dissociation. Comparing to the well-known rich picture of quasi-static thermally and pressure-induced atomistic transformations of OCs in diamonds [60,61], elementary paths of ultrafast photo-induced OC conversion remain almost unexplored.

Below, we demonstrate a number of novel diverse laser-induced atomistic transformations in different diamond environments (HPHT, IaAB), which change both OC configurations and their visible range spectral appearances, thus making them either optically visible or invisible.

### 3.1. Laser-Induced Generation of Blue-Red Luminous Centers in IaAB Diamonds: Vacancy-Mediated Conversion

**Figure 9:**
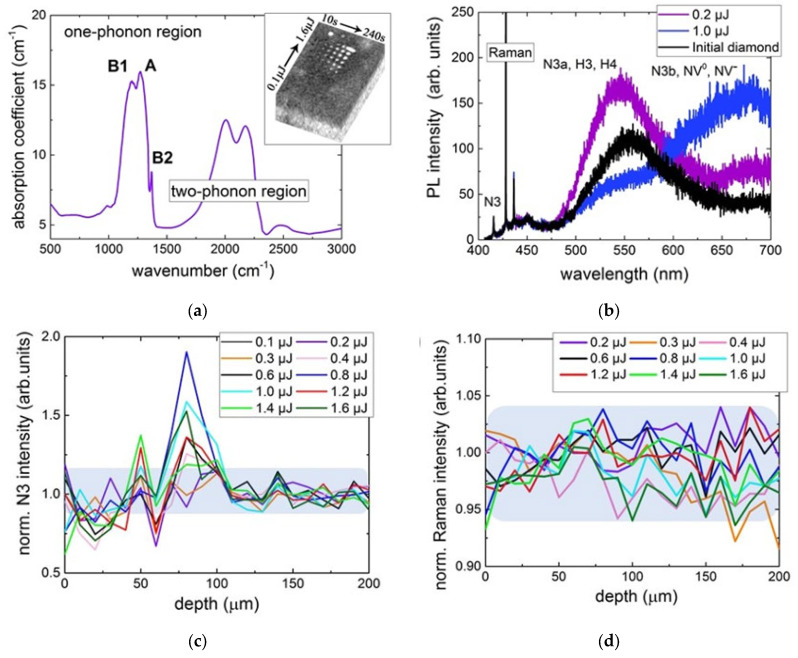
(**a**) FT-IR spectrum of the initial diamond. Inset: Array of PL micromarks in IaAB diamond inscribed by 515 nm, 0.3 ps laser pulses at 0.25-NA focusing, variable energies and exposures (×100 kHz); (**b**) Raman and PL spectra of the unexposed and laser modified regions at the selected fs-laser pulse energies of 0.2 and 1.0 µJ; (**c**) PL spectra of 415 nm N3 ZPL intensity of the unexposed and laser modified regions at different fs-laser pulse energies in the range of 0.1–1.6 µJ; (**d**) depth dependence of 428 nm Raman line intensity of the unexposed and laser modified regions at different fs-laser pulse energies in the range of 0.1–1.6 µJ.

Laser-induced non-equilibrium structural microscale modification of high-nitrogen natural diamonds plays the important role in the “stealth” gem tracing technology recently developed by PJSC ALROSA [124]. In the framework of this technology [125,126,127,128], a natural IaAB-type diamond was exposed in a series of patterns at variable energies and exposure times (Figure 9a, inset) of 515 nm, 300 fs-laser pulses focused by a 0.25-NA micro-objective at the 125-µm depth. According to FT–IR spectra, in the gemstone the average concentrations of highly aggregated nitrogen OCs equaled [A(2N)]~300 ppm, [B1(4NV)] ~500 ppm, with the minor presence of B2 (Figure 9a). Similarly, the PL spectra of the initial diamond at the 405 nm excitation exhibited the N3 (3N) band (415 nm ZPL), 1332-cm^–1^ Raman band of optical phonon in diamond (peak at 428 nm), a broad blue-green PL band of H4 (4N2V, 496 nm ZPL), H3 (2NV, 503 nm ZPL), and N3a centers (461 nm ZPL, an aggregate of N3 and interstitial-related B2 centers [129]) in the range of 476–550 nm (Figure 9b).

The exposed micromarks at the medium laser pulse energies (0.2–1.6 µJ) demonstrated the considerable increase in the intensity of 415 nm N3 PL peak (Figure 9c) but no changes in the intensity and width of the 428 nm Raman line (Figure 9d). Simultaneously, the blue-green PL band raises in the range of 476–550 nm (Figure 9b), indicating the increasing concentrations of H4 (4N2V) and H3 (2NV) and/or N3a and N3b centers (aggregates of N3 and interstitial-related B2 centers with their ZPLs at 461 and 540 nm [60,129], respectively). The appearance of the new OCs could be triggered by the two-photon generation of *I–V* pairs and occurs at the expense of the PL-blind B1 (4NV) and A (2N) precursors via the attachment of separate photo-generated vacancies (Equation (17)), present in low-concentrations:

(17) $$\begin{array}{c} C_{S}+2\hbar \omega \to C_{I}\left(I\right)+V;\\ 2N\left(A\right)+V\to 2NV\left(H3\right);\\ 4NV\left(B1\right)+V\to 4N2V\left(H4\right). \end{array}$$

The complementary photo-generated carbon interstitials *I*(*C*_I_), incidentally accumulating in the N3a,b centers [60,129] (Figure 9b) and in the carbon lattice, could compete for lattice sites with substitutional nitrogen atoms in the different OCs. This could result in the recovering of the lattice sites, *C*_S_, and generating the labile nitrogen interstitials *N*_I_ along with the observed additional 3NV (N3) or N3a,b centers:

(18) $$\begin{array}{c} C_{I}+2N\left(A\right)\to N_{S}\left(C\right)+C_{S}+N_{I};\\ C_{I}+4NV\left(B1\right)\to 3NV\left(N3\right)+C_{S}+N_{I};\\ 2NV\left(H3\right)+N_{I}\to 3NV\left(N3\right)+C_{I}. \end{array}$$

In contrast, at higher pulse energies this blue-green PL band drops in its intensity in favor of the raising yellow-red PL band (Figure 9b), representing the increasing abundance of N3b (540 nm ZPL) and NV^0^ (575 nm ZPL) centers. Their appearance could be related to either direct the photo-dissociation of the present OCs (Equation (19)), or vacancy/interstitial-mediated detachment of nitrogen atoms (Equation (20)) or both. Still, the 428 nm Raman line intensity remains constant within the minor error bars (Figure 9d) and the 415 nm N3 peak intensity increases (Figure 9c), indicating the negligible, somehow mitigated, accompanying laser damage to the carbon lattice and the accumulation of the stable N3 centers. The drop of the blue-green PL band intensity and increasing yield of N3b (aggregate of *I*-related B2 and N3 centers [60,129]) and NV^0^ center PL intensity in Figure 9b could be related to the following photo processes:

(19) $$\begin{array}{c} 4N2V\left(H4\right)+2\hbar \omega \to 2NV\left(H3\right)+2NV\left(H3\right);\\ 4N2V\left(H4\right)+2\hbar \omega \to 3NV\left(N3\right)+NV;\\ 2N\left(A\right)+2\hbar \omega \to N_{S}+N_{I}+V;N_{S}+V\to NV. \end{array}$$

Surprisingly, *I*(*C*_I_) and *V* defects could separately work in the laser-agitated lattice in similar way:

(20) $$\begin{array}{c} C_{S}+2\hbar \omega \to C_{I}+V;\\ 2N\left(A\right)+V+V\to NV+NV;\\ 2NV\left(H3\right)+V\to NV+NV;\\ 4NV\left(B1\right)+V+V+V\to NV+\mathrm{....}+NV;\\ 4NV\left(B1\right)+V\to 3NV\left(N3\right)+NV;\\ 4N2V\left(H4\right)+V+\ldots V\to NV+\mathrm{....}NV;\\ C_{I}+2NV\left(H3\right)\to NV+C_{S}+N_{I};\\ C_{I}+4NV\left(B1\right)\to 3NV\left(N3\right)+C_{S}+N_{I};\\ C_{I}+2N\left(A\right)\to N_{S}+C_{S}+N_{I};N_{S}+V\to NV;N_{I}+V\to NV+C_{I};\;C_{I}+V\to C_{S}. \end{array}$$

Overall, the ultrafast laser exposure of nitrogen OCs in the natural diamond demonstrates the strong red spectral shift of the predominating PL and related absorption band versus the fs-laser pulse energy from the blue to red spectral ranges. This indicates the apparent weak “local coloration” of the gemstone at the negligible lattice damage, underlying its “stealth” photoluminescent encoding mechanism.

### 3.2. Visible Range Laser Bleaching of HPHT Diamonds: Vacancy-Mediated Aggregation

**Figure 10:**
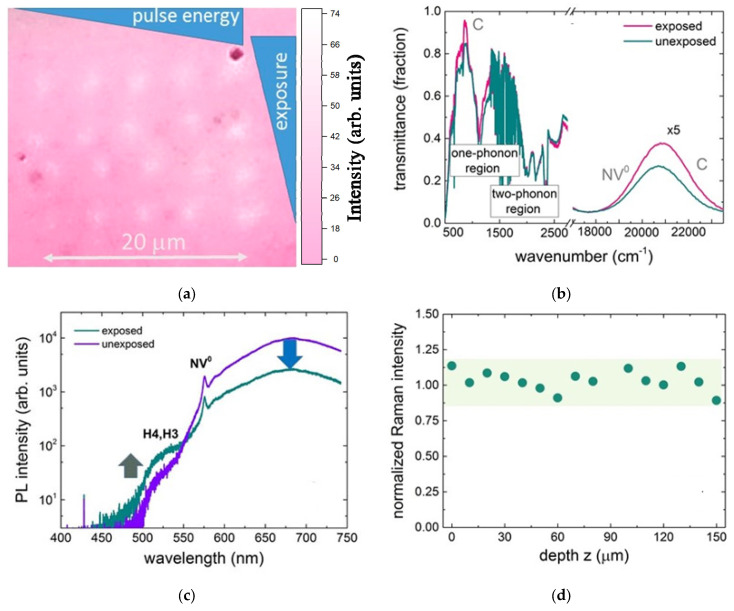
(**a**) Array of bleached micromarks in Ib HPHT diamond inscribed by 515 nm and 0.3 ps laser pulses at 0.25 NA focusing, variable energies (0.2–1.6 µJ) and exposures of 60, 120, 180, and 240 s (×100 kHz); (**b**) FT-IR and optical transmission spectra of the bleached (1.6 µJ, 240 s) and unexposed (reference) regions inside the diamond; (**c**) PL spectra of the bleached (1.6 µJ, 240 s) and unexposed (reference) regions inside the diamond; (**d**) normalized (exposed/unexposed) 428 nm Raman line intensity versus depth inside the diamond, showing no distinct damage to the carbon lattice in the bleached regions. Adapted from [120].

The alternative, bleaching process is of interest in changing ordinary intrinsic colors of natural and synthetic diamonds. Specifically, in the study, we used an Imperial Red Diamonds^TM^ crystal (LLC Velman, Russia), HPHT-produced Ib-crystal (3.5 × 2.2 × 0.9 mm^3^), red-colored after its e-beam exposure (3 MeV/5 × 1018 cm^–2^) and accompanying atmospheric pressure, high-temperature annealing in vacuum at 1200 °C for 30 min [120] (Figure 10a). The intrinsic impurity concentrations were as follows: C center ~200 ppm, NV^−^ center ~5–6 ppm, interstitial nitrogen atoms *N*_I_~15 ppm, and very minor Ni-catalyst content, with no indications of irradiation-induced neutral (GR, V^0^) or negatively charged (ND1, V^−^) vacancies in FT–IT, optical transmittance, and photoluminescence spectra (Figure 10b,c). Micromark inscription was performed by 515 nm and 300 fs-laser pulses, focused by a 0.25 NA micro-objective inside the diamond at the depth z~100 µm and at the 100 kHz repetition rate for 10, 30, 60, 120, and 240 s at variable pulse energies of 0.6, 0.8, 1.2, and 1.6 µJ, thus producing an array of a few micron-wide, visibly bleached back-lit exposed regions (Figure 10a).

As a result of the two-photon laser-induced bleaching in the visible range [120], the decrease of NV^0^ and NV^−^ center bands, excited at the 405 nm and 532 nm wavelengths, by 2–3 times in the bleached micro-regions regarding the unexposed material, was observed (Figure 10c). This accompanied by the minor increase of their PL yield at low-intensity exposures deeper (near the expected focal point) inside the sample and coincided with the enhanced transmission in the main C center and minor A center IR-absorption bands. In contrast, the considerably enhanced blue-green PL band of H3 and H4 centers, excited at the 405 nm wavelengths, was identified in the bleached micro-regions regarding the unexposed material (Figure 10c), recovering back near the expected focal point. Finally, the normalized (bleached/unexposed) 428 nm Raman line intensity appears constant within the error bars across the bleached region, indicating the absent considerable laser damage to the material (Figure 10d).

Our related analysis reveals that the structural modification of the present primitive nitrogen OCs (C and A centers) occurs via the two-photon generation of electron–hole plasma and accompanying self-trapped excitons [50,59]. Such excitons could be consequently converted into Frenkel carbon “interstitial vacancy” pairs with low concentrations at lower fs-laser intensities and high concentration at higher fs-laser intensities. Then, at lower fs-laser intensities the separate rare vacancies aggregate with the intrinsic C and A centers, resulting in NV and 2NV (H3) aggregates (Equation (21)):

(21) $$\begin{array}{c} C_{S}+2\hbar \omega \to C_{I}+V;\\ N_{S}\left(C\right)+V\to NV;\\ 2N\left(A\right)+V\to 2NV\left(H3\right). \end{array}$$

Actually, in this regime one can see the transmittance increase (bleaching) of NV^–^ (strong) and NV^0^ (weak) bands peaked at ≈610 and 530 nm [120] (Figure 10b), respectively, where the NV^–^ center could be converted into NV^0^ one just due to the laser heating and accompanying thermal ionization [130].

However, in this regime one can see the transmittance increase (bleaching) of NV^–^ (strong) and NV^0^ (weak) bands peaking at ≈610 and 530 nm [120] (Figure 10b), respectively, where the NV^–^ center could be converted into a NV^0^ center due to the laser heating and accompanying thermal ionization [130].

Furthermore, at higher fs-laser intensities one could expect either (non)linear photoexcitation and the activation of the present C centers (absorption peak at 450 nm), and of diatomic A centers to atomic nitrogen substitutional (*N_S_*, C) and/or interstitial (*N_I_*) centers. The latter could further interact with free photo-generated vacancies, forming NV centers (Equation (22)). In the same direction could work the interaction of high vacancy concentrations with separate C and A centers, forming NV centers. However, in reality, in this process there is a strong transmittance increase (bleaching) of C and NV^0^ bands peaked at ≈450 and 530 nm [120], at the saturated transmittance level of the NV^–^ band (Figure 10b). This indicates that both C and NV^0^ centers are converted into blue/UV-absorbing higher-aggregated centers (ZPL wavelengths < 420 nm, Figure 10c), e.g., into N3 centers with their ZPL at 415 nm [60], optically blind B1(4NV) centers or weakly absorbing/luminescent H3 and H4 centers with ZPLs at 496 nm and 503 nm [60]:

(22) $$\begin{array}{c} N_{S}\left(C\right)+2\hbar \omega \to N_{I}+V;\\ N_{S}\left(C\right)+N_{I}\to 2N\left(A\right)+C_{I};\\ NV+N_{I}\to 2NV\left(H3\right)+C_{I};\\ 2NV\left(H3\right)+N_{I}\to 3NV\left(N3\right)+C_{I};\\ 3NV\left(N3\right)+N_{I}\to 4NV\left(B1\right)+C_{I};\\ C_{I}+V\to C_{S}. \end{array}$$

Hence, color tuning in the HPHT Ib-diamond to strengthen its local optical bleaching could be realized via the fs-laser-induced conversion of the intrinsic C and A centers, which are highly absorbing in the visible range [60]; and into H3 and H4 centers, which weakly absorb in the blue spectral region; or N3(a) centers, highly absorbing in the blue-UV range (<400–460 nm) [60], thus enabling novel optical encoding paths in this type of diamonds.

## 4. Concluding Remarks

Our research attempted to enlighten the basic processes underlying the key enabling technology of direct ultrashort pulse laser writing in the bulk diamonds of a single-photon source, radiation sensors and integrated circuits fabrication, nanophotonics, spintronics, and microfluidics. Diverse experimental methods were used or adapted to characterize the related ultrafast phenomena, such as laser propagation at high focusing NA, non-linear photoexcitation and relaxation in electron–hole plasma, energy transfer into the crystalline lattice and stress buildup, the generation of vacancies and their role in atomistic reconfiguration, and the optical re-coloration of the present optical centers in diamond, characterizing them well for future dedicated detailed two-temperature and molecular dynamics simulations.

Much higher optical non-linearities enhanced by intermediate intra-gap optically active nitrogen impurity states were revealed in micro-filamentation and multi-photon electronic excitation studies for ultrashort laser pulses in nitrogen-doped and very pure synthetic diamonds. Sub-picosecond electronic lattice softening and anisotropic hot phonon stress buildup, accompanied by phonon–phonon relaxation and homogeneous thermal stresses, were visualized by means of in situ dynamic Raman scattering. Picosecond-scale plasma transport was shown to provide ultrafast energy dissipation on a micrometer-scale around the focal volume, increasing the heat-affected zone. The abovementioned electronic, stress generation, and thermal relaxation stages could contribute to the atomistic structural transformations of nitrogen impurity centers. Such transformations were observed in a synthetic diamond by complementary optical and vibrational micro-spectroscopy via the generation of Frenkel interstitial–vacancy pairs (without crucial lattice damage), vacancy attachment to nitrogen centers at low concentrations and detachment of nitrogen atoms by vacancies at their high concentrations. In contrast, in a natural IaAB-type diamond enriched by a multitude of various nitrogen centers, the more sophisticated chains of structural transformations in the laser focal volume could be anticipated based on photoluminescence microspectroscopy studies, underlying the “stealth” encoding technology. Comparing to other, synthetic boron-, silicon-, germanium-, and phosphorus-doped diamonds [1,2,3,4,5,6,131,132], broadly available natural diamonds provide large flexibility in the concentrations of nitrogen impurity and the atomistic paths of their photo-conversion.

Meanwhile, serious challenges are still present prior to the potential photoluminescent laser-based encoding applications in diamonds, being related to intrinsic diamond structural and chemical inhomogeneity in growth zones and sectors, the control of laser filamentation, inhomogeneous photoexcitation, and photo-conversion via multiple paths. Moreover, more detailed structural studies of photo-induced transformations in nitrogen centers, both internal or involving carbon interstitials/vacancies, are still required to be performed in thin diamond plates, enabling their comprehensive pre- and post-encoding characterization by optical and vibrational micro-spectroscopies.

Finally, our research on the fundamentals of ultrashort pulse laser inscriptions in diamonds not only delivers key laser–material interaction characteristics but also sets up an informative and facile framework for the comprehensive fundamental studies of ultrashort pulse laser writing processes in other, both crystalline and amorphous, dielectrics [133,134,135,136,137,138,139,140] to pave the way for novel technological advances.

## Data Availability

The data supporting the reported results can be obtained from the authors.
